# Transcranial pulsed ultrasound facilitates brain uptake of laronidase in enzyme replacement therapy for Mucopolysaccharidosis type I disease

**DOI:** 10.1186/s13023-017-0649-6

**Published:** 2017-06-08

**Authors:** Yu-Hone Hsu, Ren-Shyan Liu, Win-Li Lin, Yeong-Seng Yuh, Shuan-Pei Lin, Tai-Tong Wong

**Affiliations:** 1 Institute of Biomedical Engineering, College of Medicine and College of Engineering, National Taiwan University, No. 1, Sec. 4, Roosevelt Rd., Taipei, 10617 Taiwan; 2 Department of Neurosurgery, Cheng-Hsin General Hospital, Taipei, Taiwan; 3 Biomedical Imaging and Radiological Sciences, National Yang-Ming University, No.155, Sec.2, Linong Street, Taipei, 112 Taiwan; 4 National PET/Cyclotron Center, Department of Nuclear Medicine, Taipei Veterans General Hospital, Taipei, Taiwan; 5 Molecular and Genetic Imaging Core/Taiwan Mouse Clinic, National Comprehensive Mouse Phenotyping and Drug Testing Center, Taipei, Taiwan; 6 Institute of Biomedical Engineering and Nanomedicine, National Health Research Institutes, Miaoli, Taiwan; 7 Department of Pediatrics, Cheng-Hsin General Hospital, No.45, Cheng Hsin St., Pai-Tou, Taipei, 112 Taiwan; 8 Department of Pediatrics, National Defense Medical Center, Taipei, Taiwan; 9 Department of Medicine, MacKay Medical College, New Taipei City, Taiwan; 10 Department of Pediatrics, MacKay Memorial Hospital, No. 92, Sec. 2 Chung-Shan North Road, Taipei, 10449 Taiwan; 11 Department of Medical Research, MacKay Memorial Hospital, No. 92, Sec. 2 Chung-Shan North Road, Taipei, 10449 Taiwan; 12 Department of Early Childhood Care, National Taipei University of Nursing and Health Sciences, Taipei, Taiwan; 13 Division of Pediatric Neurosurgery, Neurological Institute, Taipei Veterans General Hospital, Taipei, Taiwan; 14 Institutes of Clinical Medicine, Taipei Medical University, Taipei, Taiwan; 15 Division of Pediatric Neurosurgery, Department of Neurosurgery, Taipei Medical University Hospital, Taipei Medical University, 252 Wuxing St, Taipei, 11031 Taiwan; 16 Joint Biobank, Office of Human Research, Taipei Medical University, Taipei, Taiwan

**Keywords:** Mucopolysaccharidosis type I, Blood-brain barrier, Ultrasound, Recombinant human alpha-L-iduronidase

## Abstract

**Background:**

Mucopolysaccharidosis type I (MPS I) is a debilitating hereditary disease characterized by alpha-L-iduronidase (IDUA) deficiency and consequent inability to degrade glycosaminoglycans. The pathological accumulation of glycosaminoglycans systemically results in severe mental retardation and multiple organ dysfunction. Enzyme replacement therapy with recombinant human alpha-L-iduronidase (rhIDU) improves the function of some organs but not neurological deficits owing to its exclusion from the brain by the blood-brain barrier (BBB).

**Methods:**

We divided MPS I mice into control group, enzyme replacement group with rhIDU 2.9 mg/kg injection, enzyme replacement with one-spot ultrasound treatment group, and enzyme replacement with two-spot ultrasound treatment group, and compare treatment effectiveness between groups. All ultrasound treatments were applied on left side brain. Evans blue was used to simulate the distribution of rhIDU in the brain.

**Results:**

Transcranial pulsed weakly focused ultrasound combined with microbubbles facilitates brain rhIDU delivery in MPS I mice receiving systemic enzyme replacement therapy. With intravenously injected rhIDU 2.9 mg/kg, the IDUA enzyme activity on the ultrasound treated side of the cerebral hemisphere raised to 7.81-fold that on the untreated side and to 75.84% of its normal value. Evans blue simulation showed the distribution of the delivered drug was extensive, involving a large volume of the treated cerebral hemisphere. Two-spot ultrasound treatment scheme is more efficient for brain rhIDU delivery than one-spot ultrasound treatment scheme.

**Conclusions:**

Transcranial pulsed weakly focused ultrasound can open BBB extensively and facilitates brain rhIDU delivery. This novel technology may provide a new MPS I treatment strategy.

## Background

Mucopolysaccharidosis type I (MPS I) is an autosomal recessive disorder characterized by deficiency of alpha-L-iduronidase (IDUA) and consequently inability to degrade glycosaminoglycan (GAG). The pathological accumulation of GAG systemically causes severe intellectual disability, cardiomyopathy, skeletal deformities, hepatosplenomegaly, and other organ dysfunctions. The life expectancy of patients with MPS I is less than 10 years. Enzyme replacement therapy with laronidase (a recombinant human alpha-L-iduronidase, rhIDU) has been shown to improve organ function but not produce neurological benefits owing to its lack of blood-brain barrier (BBB) penetration [1, 2].

The BBB is composed of tight junctions between capillary endothelial cells, a basement membrane, and foot processes of astrocytes. Normally only small molecules with a molecular weight less than 400 Da can pass through [3–8], and more than 98% of small molecular drugs cannot [9].

Pulsed ultrasound combined with microbubbles (MBs) has been shown to open the BBB temporarily and reversibly without causing significant tissue damage [3–8, 10–15]. With appropriate acoustic parameter settings, molecules as large as 2000 kDa can be successfully delivered to brain in the animal model [16]. Most such studies have used pulsed strongly focused ultrasound to open BBB. The characteristic of pulsed strongly focused ultrasound is to concentrate acoustic energy to a small focal zone, therefore BBB opening (BBBO) can be achieved in a small highly selective area within the brain [3–8, 10–13]. This technique is appropriate for brain tumors, for which the therapeutic agents have to be delivered to a selective area. For diseases such as MPS I, where widespread delivery of therapeutic agents to the brain is needed, the use of pulsed unfocused ultrasound to open the BBB is more suitable [14, 15, 17–19].

In our study, pulsed weakly focused ultrasound combined with MBs was used to open BBB extensively for brain laronidase delivery of MPS I mice. Evans blue (EB), which combines with albumin in plasma when injected into the circulation to form a large molecular weight conjugate with a size similar to laronidase, was used to simulate the distribution of laronidase in the brain after BBB opening.

## Methods

### Animal preparation

The animal work was carried out in accordance with the recommendations in the Guide for the Care and Use of Laboratory Animals of the National Institutes of Health (USA). The experimental protocols were approved by the Institutional Animal Care and Use Committee of Cheng Hsin General Hospital. The C57BL/6 mice (B6 mice) were obtained from the National Laboratory Animal Center; the MPS I mice (B6.129S4-Idua^tm1.1Kmke^) with Idua gene knock-in mutation on a C57BL/6 background were obtained from The Jackson Laboratory (Bar Harbor, ME, USA) and were bred and maintained by the National Laboratory Animal Center. At the time of treatment, the C57BL/6 mice had a body weight of 19–24 g, and the MPS I mice had a body weight of 25–31 g.

### Ultrasound equipment

**Fig. 1:**
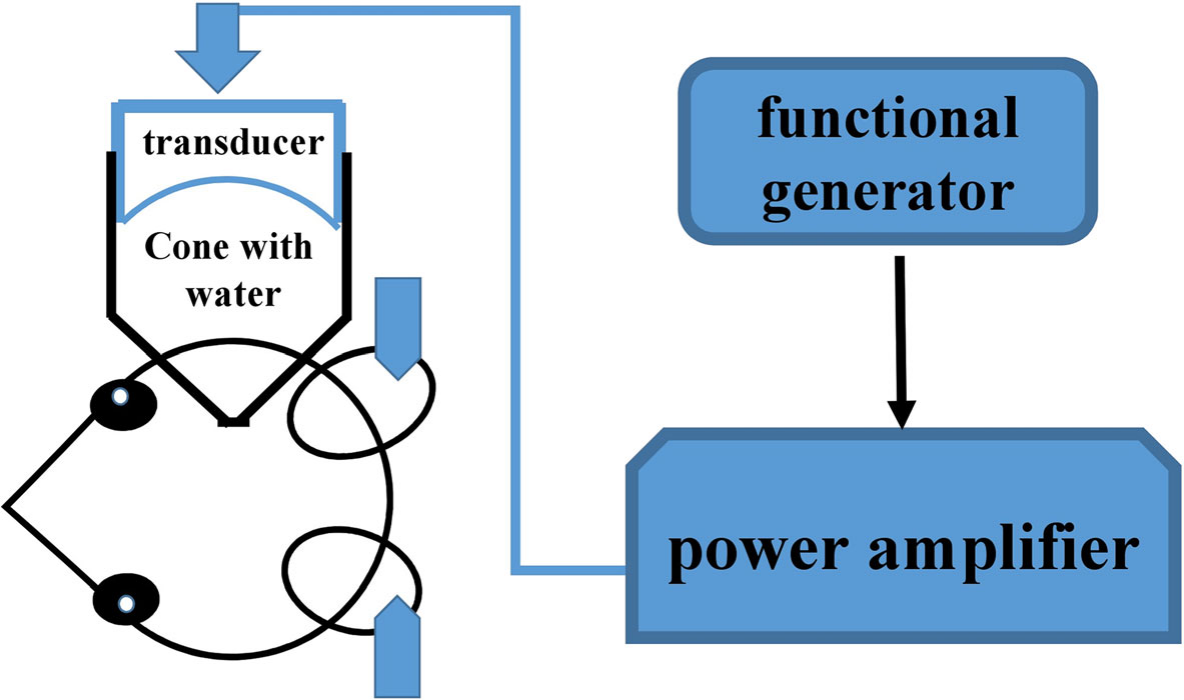
Experimental setup for BBB opening

The pulsed ultrasound was generated by a 1-MHz single element transducer (A392S, Panametrics, Waltham, MA, USA) with a diameter 38 mm and a radius of curvature 63.5 mm. A cone filled with distilled and degassed water was mounted onto the transducer, and the cone tip was capped by a polyurethane membrane. The transducer with cone water was fixed to a stereotactic apparatus (David Kopf Instruments, Tujunga, CA, USA), allowing movement of the transducer to different sonication targets, and was driven by a functional generator (33220A, Agilent Technologies, CO, USA) and a power amplifier (75A250A, Amplifier Research, Souderton, PA, USA). The mouse was in a prone position with its head beneath the cone tip. Ultrasound transmission gel was used to ensure good contact between the head and cone tip (Fig. 1).

### Experimental arrangement

The plasma pharmacokinetics of laronidase was investigated in MPS I mice injected with 2.9 mg/kg (5 times the clinical dose used in enzyme replacement therapy) intravenously. Blood samples were collected for IDUA enzyme activity assay at 0.5, 1, 2, 4, 6, 8 and 24 h after injection to find out the optimal time point for laronidase accumulation. IDUA enzyme activity of the liver was compared to that of the brain under normal circumstances.

**Fig. 2:**
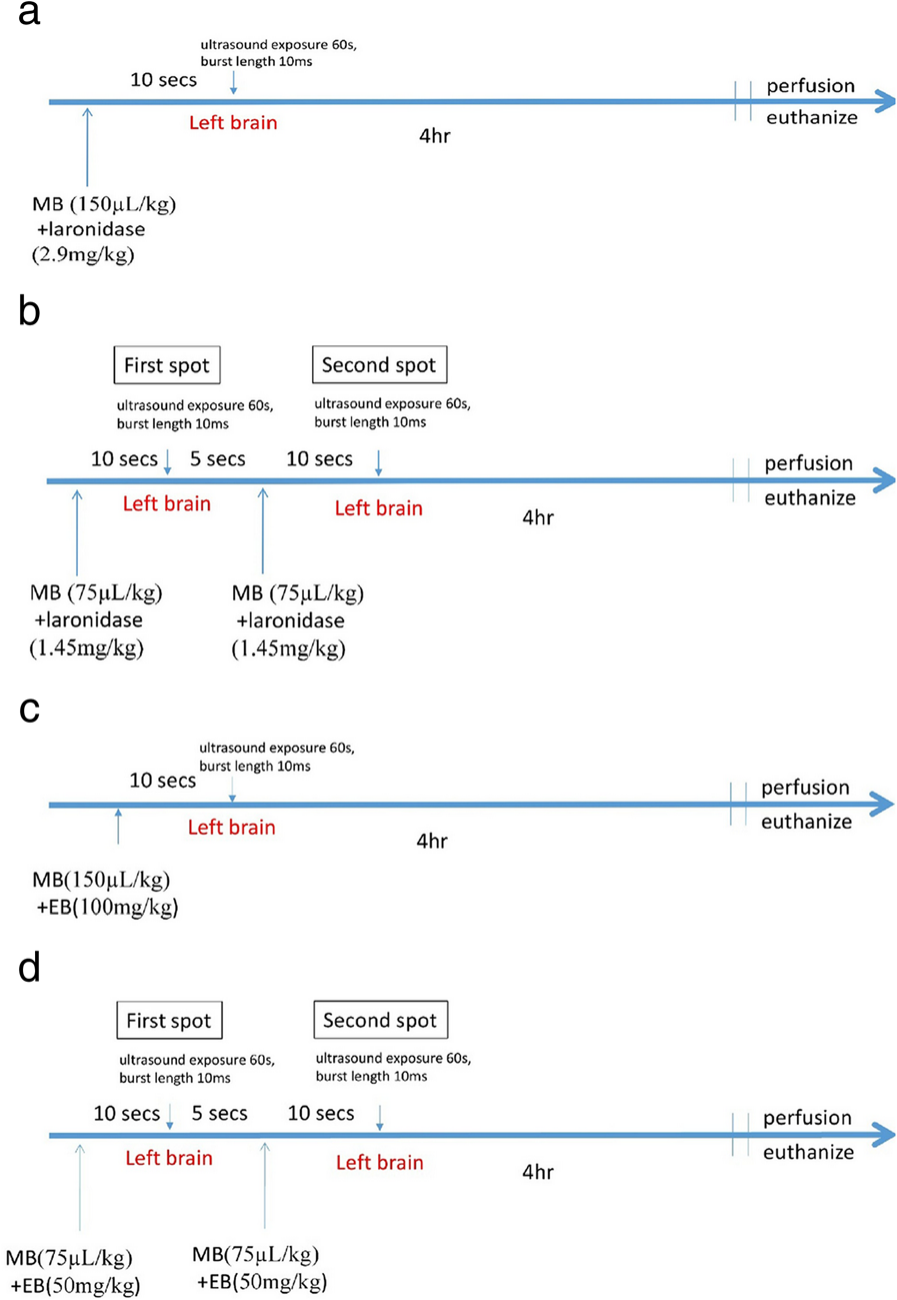
Procedures of ultrasound treatment in MPS I mice and B6 mice. Ultrasound was applied in the left side brain. Acoustic pressure 0.56 MPa, PRF 1 Hz, burst length 10 ms, and ultrasound exposure time 60s were used in all ultrasound treatments. **a** MPS I mice treated with one-spot ultrasound. **b** MPS I mice treated with two-spot ultrasound. The total dose of MBs and laronidase used were the same as panel a. **c** B6 mice treated with one-spot ultrasound. **d** B6 mice treated with two-spot ultrasound. The total dose of MBs and EB used were the same as panel **c**

To study laronidase delivery, the MPS I mice were divided into 4 groups. Group one (*n* = 3) received no treatment; group two (*n* = 4) received tail-vein injection of laronidase 2.9 mg/kg; group three (*n* = 4) received injection of laronidase 2.9 mg/kg followed by one-spot ultrasound exposure (Fig. 2a), and group four (*n* = 4) received two injections of laronidase 1.45 mg/kg (total 2.9 mg/kg), followed by two-spot ultrasound exposure (Fig. 2b). The IDUA enzyme activity in brain was compared between MPS I mice and normal B6 mice (*n* = 4) to assess therapeutic effects. In one-spot ultrasound exposure group, laronidase 2.9 mg/kg plus MBs 150 uL/kg were injected just before ultrasound exposure (acoustic pressure 0.56 MPa, pulse repetition frequency [PRF] 1 Hz, burst length 10 ms and sonication duration 60 s). In two-spot ultrasound exposure group, a total dose of MBs 150 uL/kg plus laronidase 2.9 mg/kg was injected in two half-injection, each half was injected just before each spot ultrasound exposure (acoustic pressure 0.56 MPa, PRF 1 Hz, burst length 10 ms and sonication duration 60 s for each spot). The mice were euthanized 4 h after treatment.

EB was used to simulate the distribution of laronidase in the brain. With the same acoustic parameters used in MPS I mice, the B6 mice were subjected to the BBB opening procedure, and EB 100 mg/kg instead of laronidase was delivered into the brain. The B6 mice were divided into 3 groups: group 1 (*n* = 5) received EB 100 mg/kg injection only, group 2 (*n* = 12) received EB 100 mg/kg plus MBs 150 uL/kg injection and the previously described one-spot ultrasound treatment (Fig. 2c), group 3 (*n* = 6) received two EB 50 mg/kg plus MBs 75 uL/kg injection (total EB 100 mg/kg plus MBs 150 uL/kg) and the previously described two-spot ultrasound treatment (Fig. 2d).

### Animal procedures

**Fig. 3:**
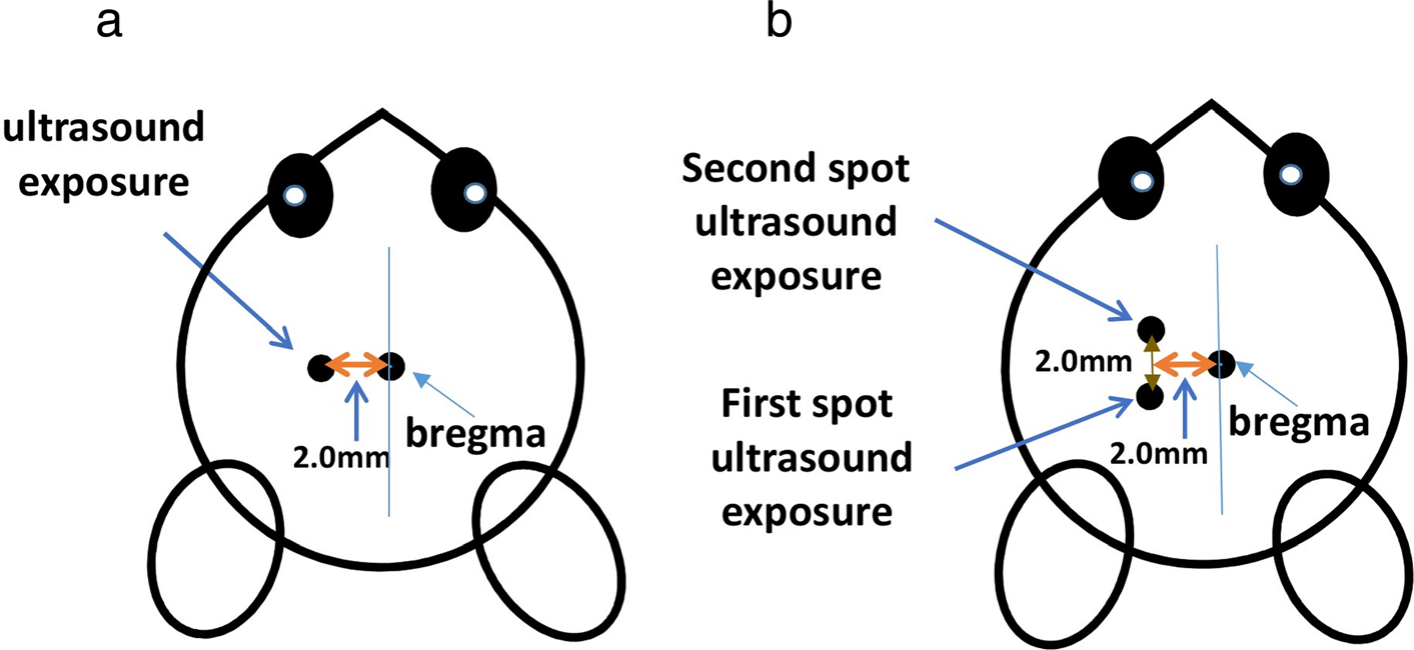
Locations of ultrasound exposure on MPS I mice and B6 mice. **a** One-spot ultrasound exposure. **b** Two-spot ultrasound exposure

Each mouse was anesthetized by intraperitoneal injection of Zoletil (20 mg/kg; Zoletil®50, Virbac Laboratories, Carros, France) plus Xylazine (5 mg/kg; Rompun™, Bayer, Shawnee Mission, KS, USA) before procedures. For mice receiving intravenous laronidase or EB injection without ultrasound treatment, the brain was perfused transcardially with normal saline 4 h after drug injection, followed by euthanasia, and then the brain was harvested and assayed for EB or laronidase.; for mice receiving intravenous laronidase or EB injection with ultrasound treatment, the mice were fixed to the stereotactic apparatus, the tail vein was catheterized. For one-spot ultrasound exposure, the cone of the ultrasound transducer was put 2 mm left lateral of the bregma (Fig. 3a); for two-spot ultrasound exposure, the cone of the ultrasound transducer was put first 1 mm posterior and then 1 mm anterior to the one-spot location (2 mm left lateral of the bregma; Fig. 3b). With the mouse fixed to the stereotactic apparatus and the cone of the ultrasound transducer in place, a solution of MBs (SonoVue®, Bracco, Amsterdam, The Netherlands) mixed with EB (Sigma-Aldrich, St. Louis, MO, USA) or MBs mixed with laronidase (Aldurazyme®, Biomarin Pharmaceutical, San Rafael, CA, USA) was injected through the tail vein and followed immediately by ultrasound exposure. Four hours after treatment, the mouse brain was perfused transcardially with normal saline to wash out the content from the cerebral vasculature, and then the brain was harvested, sliced, and assayed for EB or laronidase.

### Evans blue quantification

The mouse brain samples were weighed, placed in trichloroacetic acid solution, homogenized, and centrifuged. The extracted EB was diluted with 95% ethanol in a ratio of 1:3, then put into multiwell-plate-reading fluorometer with excitation wavelength set to 620 nm (X-Zell Biotec, Bangkok, Thailand) and absorption wavelength set to 680 nm. The fluorescence was converted to concentration using a standard curve of known EB concentrations.

### IDUA enzyme activity assay

IDUA enzyme activity was measured by a fluorometric assay. Mouse brain samples were homogenized, lysed with a lysis buffer, and centrifuged. The substrate of the IDUA, 4-methylumbelliferyl alpha-l-iduronide (4MU-I, Toronto Research Chemicals, Toronto, Ontario, Canada, #M334701), was diluted with sodium formate buffer (50 mM, pH 2.8), then 25 μL aliquots of 4MU-I (200 μM) were mixed with 25 μL aliquots of tissue homogenates. The mixture was incubated at 37 °C for 20 h, and then 200 μL glycine carbonate buffer (pH 10.5) was added to end the reaction. The intensity of emitted fluorescence resulted from enzyme-substrate reaction was measured with excitation at 365 nm and emission at 450 nm. A standard curve was made by 4-Methylumbelliferone (4MU, Sigma-Aldrich #M1381). Protein was determined by Pierce BCA protein assay kit (ThermoFisher scientific #23227). IDUA enzyme activity was expressed as units/mg protein * 20 h.

### Statistical analysis

SPSS software (IBM SPSS statistics, IBM Corp., Armonk, NY, USA) is used for statistical analysis. Data are presented as mean +/- SEM. A Tukey test was used for comparisons between paired samples, and one-way ANOVA was used for comparisons between three or more samples. *P* < 0.05 was considered statistically significant.

## Results

### Laronidase delivery to the brain of MPS I mice

**Fig. 4:**
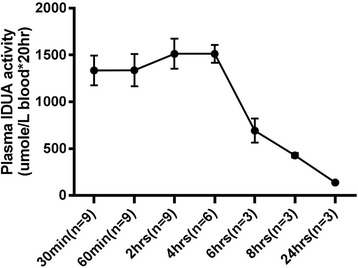
Plasma IDUA enzyme activity assay of MPS I mice. MPS I mice receiving laronidase 2.9 mg/kg injection through the tail vein, the blood were collected for IDUA activity assay at different time points after injection.

**Fig. 5:**
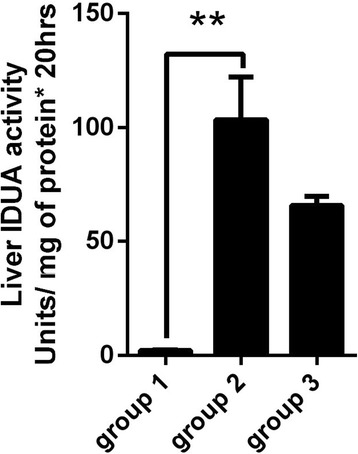
The liver IDUA activity of MPS-I and B6 mice. Group 1: MPS-1 mice without any treatment (*n* = 3). Group 2: MPS-1 mice with laronidase 2.9 mg/kg injection (*n* = 4), measured 4 hours after injection. Group 3: normal B6 mice (*n* = 4). ** indicates *p* < 0.01

**Fig. 6:**
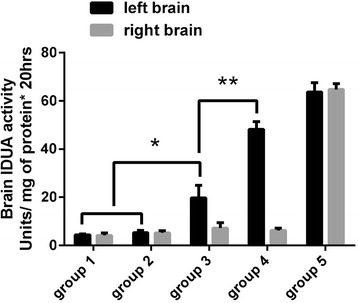
Brain laronidase delivery. Group 1 (*n* = 3): MPS I mice without any treatment. Group 2 (*n* = 4): MPS I mice receiving laronidase 2.9 mg/kg injection without ultrasound treatment. Group 3 (*n* = 4): MPS I mice receiving one-spot ultrasound exposure. MBs 150μL/kg plus laronidase 2.9 mg/kg was injected. Group 4 (*n* = 4): MPS I mice receiving two-spot ultrasound exposure. A total dose of MBs 150μL/kg plus laronidase 2.9 mg/kg was injected. Group 5 (*n* = 4): normal B6 mice without any treatment. * indicates *p* < 0.05 ** indicates *p* < 0.01

Our analysis of laronidase pharmacokinetics in MPS I mice plasma showed that the plateau of IDUA enzyme activity persisted 4 h after laronidase injection (Fig. 4), suggesting that activity in the brain and liver should be measured 4 h after treatment. Although liver IDUA enzyme activity of MPS I mice was significantly higher in those injected with laronidase 2.9 mg/kg than those without treatment (Fig. 5), brain IDUA enzyme activity was similar between the groups (Fig. 6, group 1 and group 2), indicating that the BBB blocks the entry of laronidase to the brain. For MPS I mice receiving laronidase and ultrasound treatment (Fig. 2a and b), the brain IDUA enzyme activity on the ultrasound-treated side was significantly higher than that of the control group (Fig. 6, group 3 and group 4); the brain IDUA enzyme activity on the treated side was 2.75-fold and 7.81-fold that on the untreated side after one-spot and two-spot ultrasound treatment, respectively. Compared to normal B6 mice, MPS I mice receiving one-spot and two-spot ultrasound had 30.9 and 75.8% of normal brain IDUA enzyme activity on treated side, respectively.

### Drug distribution simulated by Evans blue distribution

**Fig. 7:**
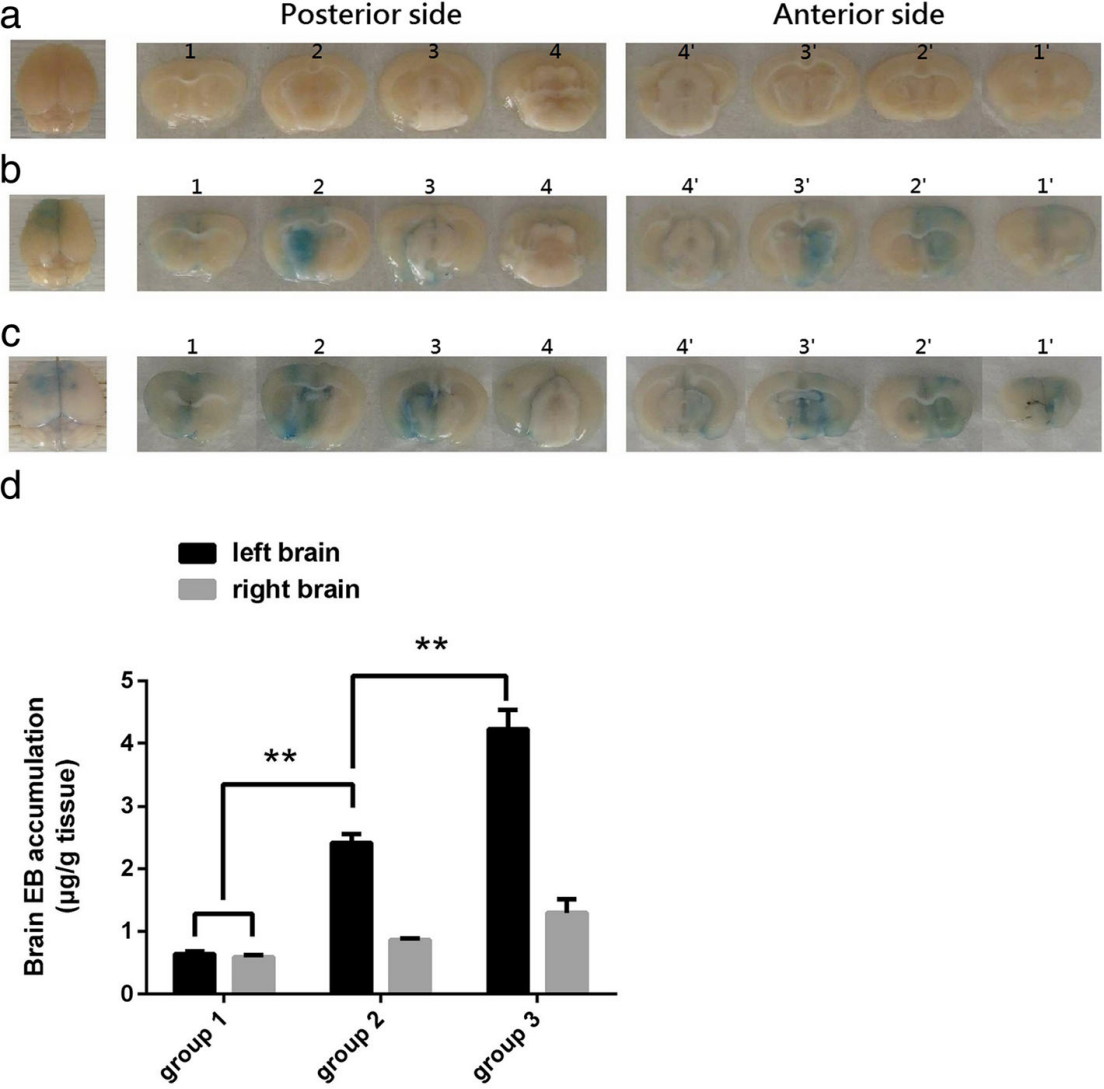
Brain EB delivery in B6 mice. **a** Brain coronal slices of the mouse receiving EB injection only (group 1). There was no significant blue stain region. Left: brain before slicing; 1 and 1’ is the posterior side and anterior side of 1st slice, respectively, and so on. **b** Brain coronal slices of the one-spot ultrasound treated mouse (group 2). Left: brain before slicing; 1 and 1’ is the posterior side and anterior side of 1st slice, respectively, and so on. **c** Brain coronal slices of the two-spot ultrasound treated mouse (group 3). Left: brain before slicing; 1 and 1’ is the posterior side and anterior side of 1st slice, respectively, and so on. **d** Brain EB accumulation in B6 mice. Group 1 (*n* = 5): B6 mice with EB 100 mg/kg injection. Group 2 (*n* = 12): B6 mice receiving one-spot ultrasound exposure. MBs 150μL/kg plus EB 100 mg/kg was injected. Group 3 (*n* = 6): B6 mice receiving two-spot ultrasound exposure. A total dose of MBs 150μL/kg plus EB 100 mg/kg was injected. ** indicates *p* < 0.01

EB was used to simulate the distribution of laronidase in the brain. The EB molecule (961 Da) binds to albumin (69 kDa) [20] in plasma to form an EB-albumin conjugate (8–14 EB molecules binds to one albumin molecule [21]), which is similar in size to laronidase (83 kDa). In B6 mice receiving ultrasound treatment for brain EB delivery (Fig. 2c and d), brain EB delivery induced by one-spot and two-spot ultrasound treatment schemes with the same acoustic parameters used in MPS I mice resulted in a diffuse pattern of EB distribution throughout the acoustic field (Fig. 7a to c). The amount of EB delivered to the brain on the treated side was 2.83-fold and 3.87-fold that on the untreated side after one-spot and two-spot ultrasound exposure, respectively (Fig. 7d).

## Discussion

Mucopolysaccharidosis type I (MPS I), also called Hurler syndrome, is an autosomal recessive disorder characterized by inability to degrade glycosaminoglycan (GAG) owing to a deficiency of alpha-L-iduronidase (IDUA). This deficiency is attributed to mutations in the IDUA gene, which is located on chromosome 4p16.3. Patients with Hurler syndrome develop skeletal deformities, hepatosplenomegaly, loss of vision and hearing, upper airway obstruction, cardiomyopathy, and severe mental retardation, and almost all patients die before they reach 10 years of age. Enzyme replacement therapy with a recombinant human alpha-L-iduronidase (laronidase) is an important treatment option for these patients. Such therapy improves lung function, walking ability, urine GAG excretion, hepatomegaly, and sleep apnea, but not CNS manifestations because the blood-brain barrier effectively blocks treatment by preventing enzyme entry [1, 2].

Efforts have been made to increase IDUA enzyme activity within the brain in the dog, cat, and mouse models of MPS I. The approaches used to overcome the BBB include intrathecal or intraventricular injection of rhIDU or adeno-associated virus vectors for IDUA gene therapy, intravenous injection of very high doses of rhIDU, and hematopoietic stem cell-mediated gene therapy [22–30]. Here we present a novel noninvasive technique of rhIDU delivery into the brain by use of pulsed weakly focused ultrasound combined with MBs.

The barrier function of the BBB is due to a three-layer structure consisting of tight junctions between capillary endothelial cells, a basement membrane, and foot processes of astrocytes. Normally only molecules of high lipid solubility or smaller than 400 Da can pass through the BBB [3–8]. The BBB thus protects the brain from damage due to toxic molecules, but this mechanism also blocks entry of most therapeutic molecules. For example, Herceptin (trastuzumab), a kind of monoclonal antibody, has been used to treat breast cancer successfully, but in cases of brain metastasis, the BBB limits its effectiveness. When Herceptin is administered intravenously, its concentration in CSF is only 0.3% of that in plasma [10].

Strategies to overcome or bypass the BBB to deliver therapeutic agents into the brain include intraventricular injection, convection enhanced delivery, modifying agents to cross BBB, carotid artery injection of mannitol or vasoactive agents, and intranasal delivery to cerebrospinal fluid (CSF). However, each of these is either too invasive or causes only limited BBB breakdown [31, 32].

Evidence shows that pulsed ultrasound combined with MBs can open the BBB transiently and reversibly without causing significant brain tissue damage. In most of these studies, strongly focused ultrasound was used to open the BBB in a small highly selective area [3–8, 10–15, 17–19]. In the present study, we used a pulsed weakly focused ultrasound transducer to open the BBB transcranially in an extended area.

The mechanism of pulsed ultrasound-induced BBBO is attributed to acoustic radiation force and the activity of MBs in acoustic field, including stable cavitation, microstreaming and inertial cavitation. The acoustic radiation force pushes the MBs against the capillary wall and disrupts the tight junctions between the endothelial cells. The MB volume oscillates in acoustic field, the tight junctions are stretched open when there is sufficient MB volume expansion, known as “stable cavitation”. The “microstreaming” formation near an oscillating MB exerts a shearing force on the capillary wall to pull apart the tight junctions between the endothelial cells. The MB could expand several times bigger than its original size and collapse suddenly, emitting shock waves that damage the capillary wall and cause blood extravasation, known as “inertial cavitation” [3, 4, 7, 8].

The effect of pulsed ultrasound-induced BBBO is transient and reversible, lasting from 2 to 6 h after sonication, depending on the acoustic parameters [4, 33–35]. The size of the BBB opening can be controlled by acoustic pressure [16, 36]. Chen et al. have demonstrated that an acoustic pressure of 0.84 MPa is sufficient to deliver dextran molecules of 2000 kDa to the mouse brain [16]. To date, the brain delivery of several large molecular therapeutic agents (such as anti-dopamine D4 receptor antibodies, Herceptin, liposome-encapsulated doxorubicin, genetically engineered viral gene vector, anti-amyloid beta antibodies and some chemotherapy agents) has been successful in animal models [3–6, 8, 10, 11, 13].

McDonnold et al. investigating the safety of focused ultrasound with MB-induced BBB opening in rabbits (acoustic pressure 0.7–1.0 MPa) and rhesus macaques (acoustic pressure 0.149 MPa) observed no significant brain tissue damage or delayed effects [37, 38]. In their study of repeated exposure to focused ultrasound with MBs in rats, Kobus et al. obtained repeated BBB opening (acoustic pressure 0.66–0.80 MPa) with no or limited brain tissue damage [9]. In their study of the safety of long-term repeated BBB opening by pulsed unfocused ultrasound (acoustic pressure 0.6–0.8 MPa) in a primate model, Horodyckid et al. found no change in cerebral glucose metabolism and no abnormal findings on electrophysiological recordings, only a few extravasated erythrocytes have been observed [15].

In this study, brain delivery of rhIDU (laronidase; an 83 kDa protein) was attempted in the MPS I mouse model. Most previous animal studies delivered rhIDU or IDUA gene-transducing viral vectors into the brain by intrathecal or intraventricular injection [22–27, 30]. Using this route, high drug concentration can be achieved in the CSF but not in brain tissue per se because the capacity of drugs to enter the brain’s extracellular space from the CSF is limited [39, 40]. Numerous studies have demonstrated that many agents normally impervious to the BBB can be delivered to the brain in a small highly selective region by use of strongly focused ultrasound with MBs [3–6, 8, 10, 11, 13]. This strategy is appropriate for delivery of therapeutic agents to a selective and focal region, such as brain tumors. However in cases of MPS I, the delivery of therapeutic agents into the brain should be diffuse, and pulsed weakly focused ultrasound with MBs to open the BBB extensively is the more suitable approach.

Our result showed that after intravenous injection of laronidase 2.9 mg/kg, IDUA enzyme activity in the livers of MPS I mice increased markedly (Fig. 5), but remained at almost control level in their brains (Fig. 6, group 1 and group 2), indicating that the BBB blocks the entry of laronidase to the brain. After exposure to ultrasound treatment, the IDUA enzyme activity in the MPS I mouse brain on the treated side was 2.75-fold that of the untreated side and reached 30.91% of the level in normal B6 mice in one-spot ultrasound treatment group; the effect increased to 7.81-fold that of the untreated side and reached 75.84% of the level in normal B6 mice in two-spot ultrasound treatment group (Fig. 6, group 3 and group 4). To simulate the distribution of laronidase in the brain, B6 mice were injected with EB and treated with the same ultrasound exposure schemes in the MPS I mice. When injected into circulation, EB (961 Da) binds to plasma albumin (69 kDa) [20] preferentially and completely to form a high molecular weight conjugate of 71–83 kDa (8–14 mole EB bind to 1 mole albumin) [21], which is similar in size to laronidase (83 kDa). As Fig. 7d shows, EB accumulation was similar on left and right side of the brain in mice receiving EB 100 mg/kg injection only, after treatment of ultrasound, the EB accumulation of treated side brain increased significantly. The EB accumulation on treated side brain was 2.83-fold and 3.87-fold that on the untreated side brain in one-spot and two-spot ultrasound exposure mice, respectively.

The ultrasound treatment procedure in our study opens BBB to an extent that admits laronidase and albumin to brain. Hassel et al. had reported albumin neurotoxicity [41]. They injected albumin to rat neostriatum and found that albumin exceeding 10 mg/ml was neurotoxic. As Fig. 7d shows, EB accumulation induced by one-spot and two-spot ultrasound exposure were 2.512 μg/g tissue and 4.224 μg/g tissue, respectively; considering every 8–14 EB molecules bind to one albumin molecule, the albumin delivered to brain by one-spot and two-spot ultrasound exposure should be 12.88 ~ 22.55 μg/g tissue and 21.66 ~ 37.92 μg/g tissue, respectively; which are far less than albumin neurotoxic level 10 mg/ml.

Figure 7 shows the area of BBBO stained by EB. Compared to the area of BBBO induced by strongly focused ultrasound [11], that induced by pulsed weakly focused ultrasound was more extensive (i.e., occupied more of the cortical surface area and thickness of the brain exposed to the acoustic field). Moreover, that induced by two-spot exposure was more extensive than that induced by one-spot exposure, this explains the quantification result of brain laronidase and EB delivery, which showed that with the same dose of drug injected systemically, two-spot ultrasound exposure made more drug accumulation then one-spot did. This simulation of drug delivery and distribution demonstrates the feasibility of using pulsed weakly focused ultrasound combined with MBs to deliver large therapeutic molecules to the brain diffusely, and may provide a new strategy for MPS I treatment.

## Conclusions

Pulsed weakly focused ultrasound combined with microbubbles can induce BBBO and thereby enhance laronidase delivery to the brain effectively and noninvasively in MPS I mice receiving enzyme replacement therapy. The area of BBBO induced by pulsed weakly focused ultrasound is extensive. Two-spot ultrasound exposure scheme is more efficient than one-spot ultrasound exposure scheme for brain drug delivery. This novel technique of overcoming the BBB may provide a new strategy for MPS I treatment.

## Abbreviations

BBB: Blood-brain barrier
BBBO: Blood-brain barrier opening
CSF: Cerebrospinal fluid
EB: Evans blue
GAG: Glycosaminoglycan
IDUA: Alpha-L-iduronidase
MBs: Microbubbles
MPS I: Mucopolysaccharidosis type I
rhIDU: Recombinant human alpha-L-iduronidase
RPF: Pulse repetitive frequency
